# Spontaneous rupture of a dissecting aneurysm in the superior rectal artery of a patient with neurofibromatosis type 1: a case report

**DOI:** 10.1186/1752-1947-7-249

**Published:** 2013-11-07

**Authors:** Katsuhiro Makino, Nao Kurita, Masayuki Kanai, Manabu Kirita

**Affiliations:** 1 Department of Emergency Medicine, Tokyo Metropolitan Police Hospital, Nakano 4-22-1, Nakano-ku, Tokyo 164-8541, Japan; 2 Department of Emergency Medicine, Kakogawa West City Hospital, Yonedachohiratsu 384-1, Kakogawa-shi, Hyogo 675-8611, Japan

**Keywords:** Neurofibromatosis type 1, Transcatheter arterial embolization, Vasculopathy, Von Recklinghausen’s disease

## Abstract

**Introduction:**

Neurofibromatosis type 1 is an autosomal dominant disease primarily characterized by cutaneous café au lait spots, benign cutaneous neurofibromas, tumors of the central and peripheral nervous system, multiple skeletal abnormalities, and vascular abnormalities.

**Case presentation:**

Here we describe the case of a 39-year-old Japanese man with neurofibromatosis type 1 complicated by the rupture of a dissecting aneurysm in his superior rectal artery. Our patient presented with temporary loss of consciousness and acute abdominal pain. Hemorrhagic shock and anemia were diagnosed based on a physical examination and laboratory investigations, and rapid infusion of Ringer’s lactate solution was initiated. Contrast-enhanced abdominal computed tomography revealed hemorrhagic ascites with effusion of radiopaque dye into his pelvic cavity. A ruptured aneurysm was suspected in his superior rectal artery and selective angiography of the inferior mesenteric artery confirmed this diagnosis. Transcatheter arterial embolization was successfully performed and our patient was discharged 15 days after admission, with good recovery of his hemoglobin level, and no further hemorrhage or abdominal pain.

**Conclusion:**

This case of spontaneous rupture of a dissecting aneurysm in the peripheral blood supply strongly suggests the involvement of multiple blood vessel abnormalities in neurofibromatosis type 1. Patients with neurofibromatosis type 1 should undergo regular review given their overall health and the risk of fatality in vasculopathy associated with this disease.

## Introduction

Neurofibromatosis type 1 (NF-1), also known as von Recklinghausen’s disease, is a common genetic disorder affecting approximately 1 in 3000 individuals [1]. It is an autosomal dominant disease with a varying phenotype, but its cardinal features include multiple café au lait spots, axillary and inguinal freckling, multiple discrete dermal neurofibromas, and iris hamartomas (Lisch nodules). NF-1 is frequently accompanied by vascular abnormalities in medium to large arteries, the most common being aneurysms and stenoses of the aorta, renal arteries and mesenteric vessels [2]. Most vascular lesions develop by the age of 50 years [3]. The pathophysiology of vascular change is characterized by fibromuscular dysplasia and a thickening of the tunica intima of vessel walls [4-6]. These abnormalities may result in spontaneous arterial rupture, which is a potential fatal complication.

Here were present a case of NF-1 presenting with the spontaneous rupture of a dissecting aneurysm in the superior rectal artery, that was successfully treated by transcatheter arterial embolization.

## Case presentation

A 39-year-old Japanese man presented at our hospital with temporary loss of consciousness and acute abdominal pain. His medical history included NF-1, but his family history was unremarkable, and prior to this presentation he was apparently healthy and asymptomatic.

On direct questioning, he complained of diffuse abdominal pain without signs of peritoneal irritation. On physical examination, he was cold and clammy, appeared agonal, and had conjunctival anemia; additionally, he had many café au lait spots on his body, especially on his trunk. His baseline observations were recorded as follows: temperature 35.1°C, blood pressure 66/42mmHg, pulse 126 beats/min, respiratory rate 30 breaths/min, and oxygen saturation in room air 84%. Laboratory findings revealed a hemoglobin level of 12.3g/dL with elevated serum glucose and ammonia levels. No clotting abnormalities were detected, except for antithrombin III. Analysis of his arterial blood gas showed a pH of 7.132 and a lactate level of 13.1mmol/L. A diagnosis of hemorrhagic shock was made and rapid infusion of Ringer’s lactate solution was initiated.

**Figure 1:**
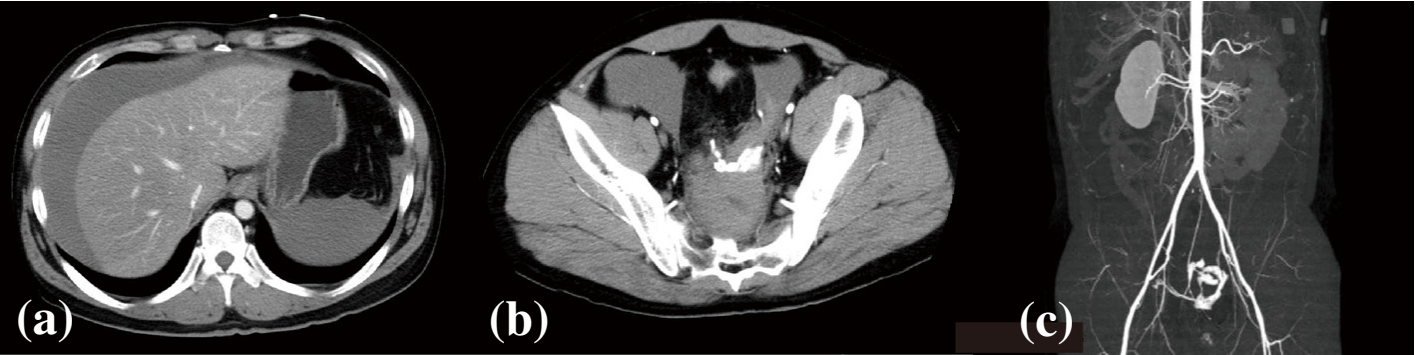
**Contrast-enhanced abdominal computed tomography on admission. (a, b)** Accumulation of hemorrhagic ascites and extravasation of radiopaque dye into the pelvic cavity. **(c)** A three-dimensional reconstruction of the computed tomographic image.

**Figure 2:**
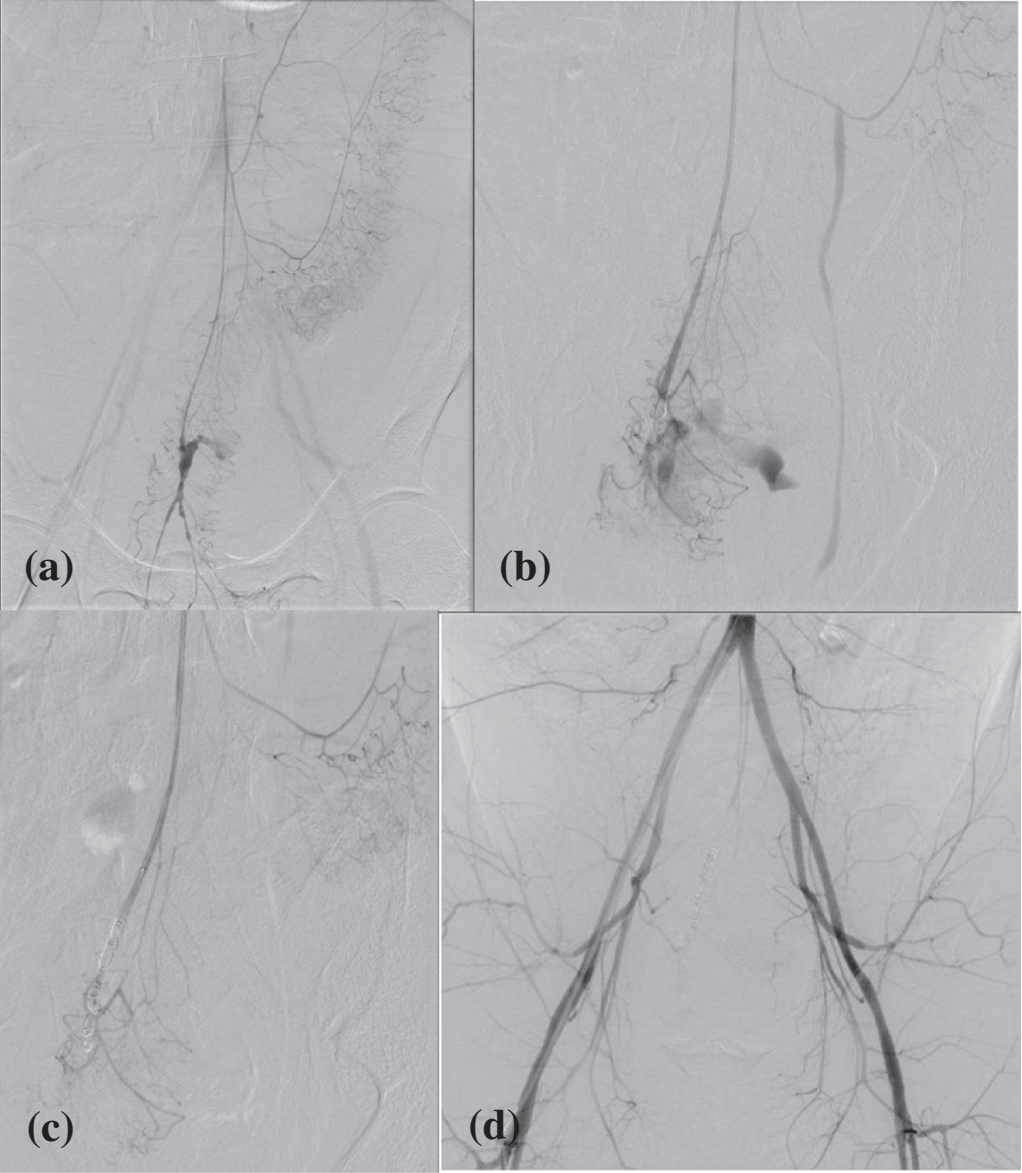
**Selective angiography of the inferior mesenteric artery. (a)** Dilatation of a distal lesion with extravasation of blood, suggesting a ruptured dissecting aneurysm in the superior rectal artery. **(b, c)** Transcatheter arterial embolization proximal to the dilatation using micro coils. **(d)** A postoperative angiographic image.

Contrast-enhanced abdominal computed tomography revealed an accumulation of hemorrhagic ascites, with effusion of radiopaque dye into his pelvic cavity. The provisional diagnosis was a ruptured vascular anomaly in his superior rectal artery, which is a branch of the inferior mesenteric artery (Figure 1). Subsequent selective angiography of his inferior mesenteric artery revealed a dilated distal lesion with extravasation of blood, and transcatheter arterial embolization was performed proximal to the dilatation using micro coils (Figure 2).

**Figure 3:**
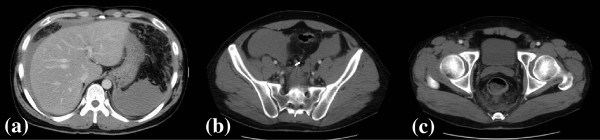
**Follow-up contrast-enhanced abdominal computed tomography taken on hospitalization day eight. (a)** Decrease in the volume of hemorrhagic ascites **(b)** An absence of extravasation **(c)** Increased contrast medium uptake by the rectal walls.

The acute hemorrhage and the fluid infusion resulted in our patient’s hemoglobin level decreasing to 4.1g/dL after treatment. His shock index was 1.90 (calculated as pulse divided by systolic blood pressure), implying that the hemorrhage volume was approximately 2L. As a result, he received red blood cell and fresh frozen plasma transfusions. Contrast-enhanced abdominal computed tomography performed after eight days of hospitalization revealed a decrease in volume of the hemorrhagic ascites, an absence of extravasation, visible rectal arteries peripheral to the embolized lesion, and increased contrast uptake by the rectal walls (Figure 3). No other vessel abnormalities were discovered in any of his major arteries from his head to the trunk of his body on either computed tomography or magnetic resonance angiography. Our patient was discharged 15 days after admission, with good recovery of his hemoglobin level, and no further hemorrhage or abdominal pain.

## Discussion

NF-1 is an autosomal dominant disorder affecting 1 in 3000 individuals, and is characterized by pigmented skin lesions (café au lait spots), cutaneous neurofibromas, tumors of the central and peripheral nervous system, and multiple skeletal abnormalities including scoliosis, local gigantism, subperiosteal bone cysts and tibial pseudarthrosis [1].

Vascular abnormalities are also considered important clinical manifestation of NF-1. Often patients with NF-1 have vascular abnormalities involving multiple vessels, however they are usually asymptomatic [5], and symptoms typically occur in childhood or early adulthood (<50 years old). Although the prevalence of vascular lesions is difficult to determine, it has been reported in the region of 0.4% to 6.4% in large clinical series [7-9]. Spontaneous arterial rupture in association with NF-1 has been described in almost every medium to large artery, including the aorta, as well as the subclavian, mesenteric and vertebral arteries. However, involvement of the renal arteries is most common [4,5]. NF-1 vasculopathy of the cerebrum, endocrine system, gastrointestinal tract and heart have also been reported [8]. Aneurysms, stenoses, arteriovenous abnormalities, arterial compression by tumors and tumor invasion have each been reported as types of vascular abnormality associated with NF-1.

The pathophysiology of vascular changes in patients with NF-1 is poorly understood, but fibromuscular dysplasia with prominent thickening of the tunica intima has been reported as a result of smooth muscle cell proliferation [10-12]. This leads to thinning of the media, weakening of the elastic lamina and narrowing of the lumen. The *NF-1* gene is large and spans over 350 kilobases; the protein product, neurofibromin, acts as a negative regulator in the Ras pathway. Neurofibromin expression has been identified in the endothelial and smooth muscle cells of blood vessels. Patients with NF-1 are usually expected to exhibit haploinsufficiency of neurofibromin activity, which may lead to cellular proliferation [8]. These findings imply a pathogenic relationship between vascular lesions and the neurofibromas that characterize NF-1.

The role of routine vascular screening in patients with NF-1 has not been evaluated. However, clinically significant lesions are relatively uncommon; therefore, periodic vascular assessment should not be recommended for all patients with NF-1 [2]. If a vascular abnormality is identified on initial screening (for example, renal ultrasound imaging for hypertension) or noninvasive imaging (for example, computed tomography angiography or magnetic resonance angiography), a detailed examination of the entire vascular system is justifiable because of the risk of multiple lesions.

Treatment is dependent on the patient’s age, as well as the type and location of the lesion. In our case, transcatheter arterial embolization was selected because it is minimally invasive and allowed rapid identification of the responsible vessel and cardiorespiratory stabilization. However, it is important to consider that this treatment is associated with a risk of acute intestinal ischemia.

## Conclusions

This case of spontaneous rupture of a dissecting aneurysm in the peripheral blood supply strongly suggests the involvement of multiple blood vessel abnormalities in NF-1. Patients with NF-1 present with a wide spectrum of vascular lesions; therefore, treatment should be selected after evaluating the patient’s general health status. Additionally, these patients should be carefully followed, taking into account that vasculopathy associated with NF-1 may lead to a fatal outcome.

## Consent

Written informed consent was obtained from the patient for publication of this case report and any accompanying images. A copy of the written consent is available for review by the Editor-in-Chief of this journal.

## Abbreviation

NF-1: Neurofibromatosis type 1.

## Competing interests

The authors declare that they have no competing interests.

## Authors’ contributions

KM and NK followed the patient, collected the data, reviewed the literature, collected all information and wrote the manuscript. MKa and MKi contributed to patient management and data collection. All authors read and approved the final version of the manuscript.
